# Establishment of a forward primers-superposed amplification analysis for accurate aspirin pharmacogenomic measurement

**DOI:** 10.1038/s41598-024-51458-0

**Published:** 2024-01-09

**Authors:** Chun-Yan Li, Ping Yang, Jie Zheng, Jing Zhang, Yi-Qing Liu, Xiao-Quan Liu, Yue Hu, Wen-Jun Lan

**Affiliations:** 1 Institute of Biomedical Engineering, Qilu University of Technology (Shandong Academy of Sciences), No.3501, Daxue Road, Jinan, 250353 China; 2 Department of Laboratory Medicine, Shandong Provincial Hospital Affiliated to Shandong First Medical University, Jinan, 250000 China

**Keywords:** Diagnostics, Pharmacology

## Abstract

Genotyping of gDNA rs12041331 (PEAR1), rs6065 (GP1BA), and rs730012 (LTC4S) can provide systematic guidance on the use of aspirin. However, an accurate, reliable and economical approach to simultaneous detection of the above single nucleotide polymorphisms (SNPs) is not reported. Herein, we designed and substantiated an allele-specific (AS) forward primer-superposed amplification analysis for measurement of the SNPs in PEAR1, GP1BA and LTC4S genes, in which the values of ∆Cq (differences in threshold cycles between the wild-type forward primer-based assay and the mutated-type forward primer-based assay) were employed to decide genotype. Mismatch AS forward primers were screened with the singleplex amplification analysis. Moreover, Cq extension optimized by AS forward primer superposition was observed in the selected forward primer-based triplex analysis. Further, robustness assessment of the triplex analysis showed the amplification efficiency ranging from 0.9 to 1.1. Precision test demonstrated the coefficient of variation of less than 2%. And the detective results of 189 DNA samples was completely concordant with that of commercial Sanger sequencing. In summary, we developed a simple, accurate and economical approach to genotyping of rs12041331 (PEAR1), rs6065 (GP1BA) and rs730012 (LTC4S) to provide a valuable pharmacogenomics tool for guidance of aspirin delivery.

## Introduction

Cardiovascular diseases, consisting of hypertension, hyperlipidemia, coronary heart disease, atherosclerosis, etc., have become the top killers of human health^1–3^. The prevention and treatment of cardiovascular diseases are increasingly becoming a focus of attention in medical field. Because of multifaceted causes and manifestations, the control of cardiovascular disease demands the use of compound formulation, commonly called polypills, which can ominously decrease the incidence of cardiovascular diseases^4^, especially cerebral apoplexy and myocardial infarct, by 35–50%^5–8^. The components of the polypill comprise statins, aspirin and a kind of antihypertensive drug^9^. The polypill concept is increasingly being recognized and accepted in clinical practice.

As a platelet aggregation antagonist, aspirin is widely used to decline the risk of suspected patients with acute myocardial infarction, and prevent cardiovascular diseases such as recurrence of myocardial infarction, stroke and atherosclerosis^10–12^. In vivo, aspirin can inactivate cyclooxygenase (COX) by irreversible acetylating the hydroxyl group of serine residues in the active part of COX^13–15^. This impedes platelet aggregation through constraining both the metabolism of arachidonic acid (AA) and the production of thromboxane A2 (TXA2)^13,16–18^. Aspirin resistance (AR) and adverse reactions were found in some patients who took routine dose of aspirin^19^. AR is defined as a lower-than-expected inhibition effect of aspirin on platelet aggregation, raising the risk of recurrent cerebral infarction and other cerebrovascular events^20–23^. Clinic studies exhibited a 5–60% incidence of AR and a 1.5% frequency of aspirin hypersensitivity causing allergy and hemorrhage in patients with cardiovascular disease^24–26^.

Aspirin pharmacogenomics evidenced that genetic polymorphism of PEAR1 (rs12041331), GP1BA (rs6065) and LTC4S (rs730012) is associated with AR and adverse reactions^27–31^. In 2020, a scoring table for genotyping of gDNA rs12041331 (PEAR1), rs6065 (GP1BA) and rs730012 (LTC4S) was proposed to provide systematic guidance for aspirin administration^32^. However, an accurate and reliable approach to simultaneous examination of the above single nucleotide polymorphisms (SNPs) is not reported. Herein, we designed and substantiated an allele-specific (AS) forward primer-superposed amplification analysis for discrimination of the SNPs in PEAR1 (rs12041331), GP1BA (rs6065) and LTC4S (rs730012) genes. The results insinuated that this analysis is a valuable tool to facilitate personalized antiplatelet therapy.

## Materials and methods

### Design strategy

First, mismatch AS F-primers were screened with singleplex amplification analysis. Next, the selected F-primers-based triplicate analysis was optimized by F-primer superposition to avoid undetermined results. Then, the optimized analysis was validated by robustness assessment and precision evaluation, as well as agreement analysis compared with Sanger sequencing. The values of ∆Cq (differences in threshold cycles between the wild-type F-primer-based amplification assay and the mutated-type F-primer-based amplification assay) were calculated to decide the outcomes.

### DNA extraction from buccal swab

The human buccal swab samples used in this study involved 189 Chinese volunteers, which was not a train set classified by aspirin resistance. This study was approved by the Biomedical Research Ethic Committee of Shandong Provincial Hospital (No.2023-417) and has been conducted in accordance with ethical standards and guidelines of the Biomedical Research Ethic Committee of Shandong Provincial Hospital. Authors of this work extend a statement assuring that this work was conducted in accordance with the Declaration of Helsinki and obtained informed consent from all participants. Genomic DNA was extracted by using the QIAamp DNA Mini kit (Cat No. 51304, QIAGEN, Dusseldorf, Germany), and the procedure was carried out according to the instructions. DNA concentration was examined with a NanoPhotometer P360 (Implen GmbH, Munich, Germany). The quality was determined by using OD 260 / 280 ratio. Sanger sequencing was conducted by Personal Biotechnology Co., Ltd (Qingdao, China).

### Primers and probes

AS F-primers, reverse primers and hydrolysis probes were designed using Primer Express 3.0, based on the information of the whole gene sequence. The second or fifth mismatch base was introduced at the 3′ end of F-primers, which were screened in a subsequent process. Probes for rs12041331 (PEAR1), rs6065 (GP1BA) and rs730012 (LTC4S) were labelled at the 5′ end with the fluorescent dye FAM, VIC, and NED, respectively, and at the 3′ end with the quencher BHQ1, BHQ1, and BHQ3, respectively. The oligonucleotide was synthesized by Sangon Biotech (Shanghai, China).

### Real- time amplification assay

Triplex amplification analysis (TaqMan qPCR) was executed in a total of 20 μL reaction mixture, which contained 10 μL AceQ® Universal U^+^ Probe Master Mix V2 (Vazyme, Nanjing, China), 0.2 μM of each wild/mutated-type F-primer, 0.2 μM of each reverse primer, 0.1 μM of hydrolysis probe and 10 ng DNA template. The singleplex amplification analysis was conducted according to the same protocol. A robotic liquid handling workstation (epMotion 5075 vt, Germany) was utilized to dispense the mix. The reaction protocols started with a contamination digestion step for 2 min at 37℃ and a pre-denaturation step for 5 min at 95 ℃, followed by 45 cycles of 95 ℃ for 10 s, and 60 ℃ for 35 s. Fluorescence data were collected at 60 ℃. These amplifications were performed on the ABI7500 Real-Time PCR Instrument (ThermoFisher Scientific Inc., MA, USA).

### Data analysis

GraphPad Prism software version 9.5 (GraphPad Software, Inc., San Diego, CA) was used to conduct data analysis and graphing.

## Results and discussion

### Screening of mismatch AS F-primers by singleplex amplification analysis

AS F-primers with a second or fifth mismatch base at 3' terminus were screened by detection of homozygote/heterozygote using singleplex real-time amplification analysis (10 ng DNA/test). And ΔCq (differences in threshold cycles between the wild-type F-primer-based amplification assay and the mutated-type F-primer-based amplification assay) was utilized to determine genotype. The principle for selection of the F-primer is the following: (a) no undetermined result was observed. (b) the Cq value was approximately 35 when wild homozygotes were detected in mutated-type F-primer-based amplification assay or when mutated homozygotes were measured in wild-type F-primer-based amplification assay. The original Cq values obtained from singleplex amplification analysis were shown in Table 1. And the selected sequences were shown in Table 2.

**Table 1:** Cq values of singleplex amplification analysis.

Genetic polymorphism	residue mismatch loci at 3' end of F-primer	Base substitution	F-primer based assay	Specimen genotype		
				wild-type	heterozygote	mutated-type
PEAR1, rs12041331	second	C > T	W	25.97 ± 0.075	27.04 ± 0.01	35.23 ± 0.33
			M	39.85 ± 0.34	26.95 ± 0.05	25.48 ± 0.01
		C > G	W	27.10 ± 0.02	28.13 ± 0.02	39.12 ± 0.94
			M	undetermined	27.42 ± 0.19	25.99 ± 0.07
	fifth	T > C	**W**	**25.83 ± 0.03**	**26.97 ± 0.01**	**33.58 ± 0.07**
			M	35.16 ± 0.22	27.09 ± 0.04	25.60 ± 0.08
		T > G	W	25.91	26.99 ± 0.03	32.04 ± 0.07
			*M*	*34.60* ± *0.22*	*27.09* ± *0.04*	*25.51* ± *0.04*
GP1BA, rs6065	second	A > C	**W**	**27.07 ± 0.09**	**28.26 ± 0.04**	**38.92 ± 0.04**
			M	undetermined	27.94 ± 0.06	33.45 ± 0.17
		A > G	W	26.60 ± 0.02	27.58 ± 0.08	undetermined
			M	38.89 ± 0.03	27.77 ± 0.06	33.19 ± 0.02
	fifth	C > T	W	26.09 ± 0.02	27.17 ± 0.04	undetermined
			M	37.24 ± 0.16	27.21 ± 0.07	33.03 ± 0.01
		C > G	W	26.07 ± 0.01	27.07 ± 0.02	undetermined
			*M*	*35.99* ± *0.07*	*27.05*	*32.50* ± *0.16*
LTC4S, rs730012	second	C > T	**W**	**24.83 ± 0.01**	**26.08 ± 0.13**	**35.16 ± 0.71**
			M	undetermined	26.14 ± 0.10	24.52
		C > G	W	25.02 ± 0.11	26.38	undetermined
			M	undetermined	26.16 ± 0.06	24.48 ± 0.04
	fifth	G > T	W	24.79 ± 0.01	26.03 ± 0.01	undetermined
			*M*	*32.67* ± *0.18*	*26.17* ± *0.05*	*24.45* ± *0.03*
		G > C	W	26.19 ± 0.02	27.43 ± 0.01	undetermined
			M	undetermined	27.17 ± 0.09	25.61 ± 0.01

W, wild-type; M, mutated-type.

Bold: selected wild-type F-primer. Italic: selected mutated-type F-primer.

**Table 2:** Sequences of primer and probe.

Genetic polymorphism	Primer sequences (5′–3′)	Probe sequences (5′–3′)
PEAR1, rs12041331	wild-type F-primer: TTCTGCTGTCTCACCTCCG	FAM-CTGCTCCCCTAGAGCCCACACTC-BHQ1
	mutated-type F-primer: CTTCTGCTGTCTCACGTCCA	
	R-primer: CTCACTGTGCCCCAACC	
GP1BA, rs6065	wild-type F-primer: CCCCAGGGCTCCTGCC	VIC-ACAACAACTTGACTGAGCTCCCCGC-BHQ1
	mutated-type F-primer: CCCCAGGGCTCGTGAT	
	R-primer: GAGATTCTCCAGCCCATTC	
LTC4S, rs730012	wild-type F-primer: CAGCCTGGATGGGGATA	NED-AGGTGGGTGGAGGAGTTAGCCGGG-BHQ2
	mutated-type F-primer: GCCTGGATGGTGACC	
	R-primer: ATGCTGGAGCCAGCCC	

PCR-based analysis is a convenient tool to discriminate SNPs. Genetic polymorphism specific-binding molecules in PCR-based analysis comprise dsDNA-binding dye, AS probe and primer^33,34^. The dsDNA-binding dye-based high-resolution dissolution curve (HRM) assay needs specific equipment module. Besides diseconomy, it is time-consuming and laborious to discover appropriate Minor Groove Binder (MGB) probe^31,35^. For enhancement of AS primer specificity, base mismatch is more economic than locked nucleic acid (LNA) decoration^32^. In present study, the mismatch AS primers as polymorphism specific-binding molecules were screened and utilized to discriminate homozygotes/heterozygotes for rs12041331 (PEAR1), rs6065 (GP1BA) and rs730012 (LTC4S).

### Development of triplex amplification analysis optimized by F-primer superposition

**Figure 1:**
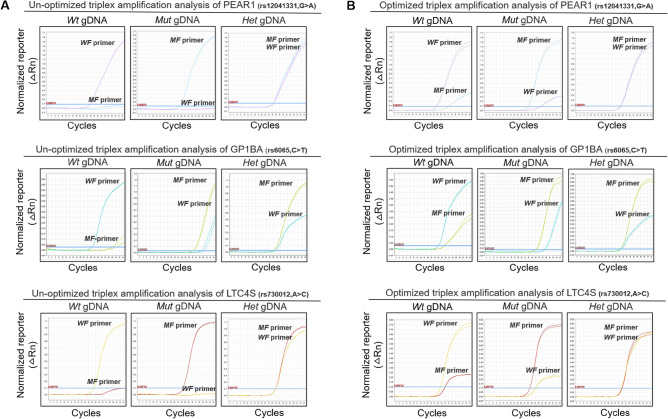
Amplification plots of the triplex amplification analysis. The wild-type F-primer-based amplification assay and the mutated-type F-primer-based amplification assay were used to detect wild-type, mutated-type and heterozygous gDNA. (**A**) Amplification plots of un-optimized triplex amplification analyses. (**B**) Amplification plots of optimized triplex amplification analyses. Represented amplification plots are shown. The reaction was run in duplicate, in which 10 ng genomic DNA was inputted. *Wt* gDNA, wild-type genomic DNA; *Mut* gDNA, mutated-type genomic DNA; *Het* gDNA, heterozygote genomic DNA; *WF* primer, wild-type forward primer; *MF* primer, mutated-type forward primer.

Based on the selected mismatch AS F-primers, we developed a triplex amplification analysis optimized by F-primer superposition. Undetermined results were observed when homozygotes were measured by un-optimized triplex amplification analysis (Fig. 1A**)**. The extension of Cq was forced by the mismatch AS F-primer superposition, which was implemented with the addition of 0.01 μM mutated/wild-type F-primer into 0.2 μM wild/mutated-type F-primer-based amplification assay (Fig. 1B**)**. The results showed that all outcomes of homozygotes were positive, suggesting that the mismatch AS F-primer superposition can improve detective convenience via omitting positive controls in the triplex amplification analysis.

### Robustness assessment of triplex amplification analysis

**Figure 2:**
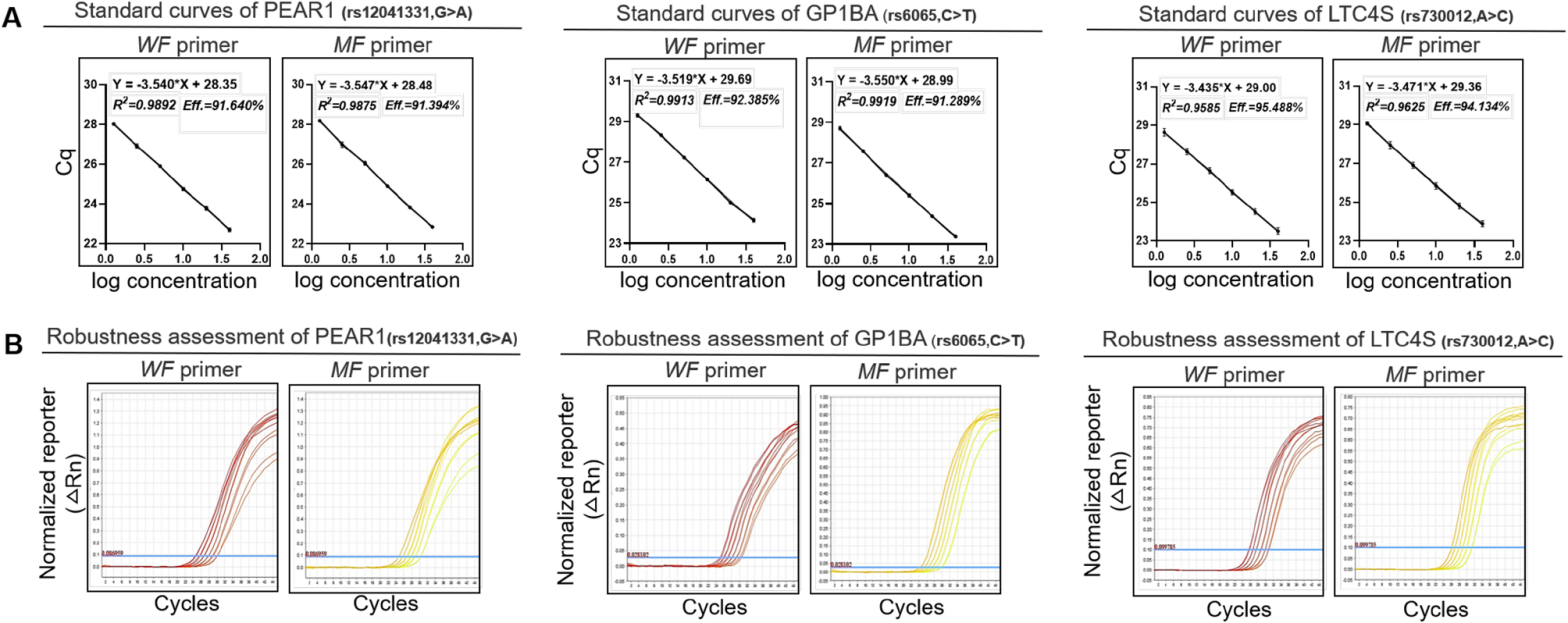
Robustness assessment of the triplex amplification analysis. The robustness assessment was executed by employing mismatch allele-specific forward primers targeting single nucleotide polymorphism to simultaneously detect heterozygotes for PEAR1 (rs12041331), GP1BA (rs6065) and LTC4S (rs730012). Serial dilutions of heterozygote (1.25–40 ng) were measured by the triplex amplification analysis. Reactions were run in duplicate with three independent experiments. (**A**) Standard curve of the triplex amplification analysis. Amplification efficiency (Eff) % and R^2^ are shown. Data are expressed as mean ± SE. (**B**) Amplification plots of the robustness assessment. Represented amplification plots are shown. *WF* primer, wild-type forward primer; *MF* primer, mutated-type forward primer.

To assess robustness of the triplex amplification analysis, the heterozygote was gradually reduced to generate gDNA samples at levels of 40 ng, 20 ng, 10 ng, 5 ng, 2.5 ng and 1.25 ng. Reactions were run in duplicate with three independent experiments. We used the following formula to calculate the amplification efficiency: 10^−1/slope ^− 1, when the logarithm of the template concentration was plotted on the *x-*axis and Cq was plotted on the *y-*axis. The results demonstrated that the amplification efficiency calculated from standard curve ranged from 0.9 to 1.1 (Fig. 2), and limit of detection (LOD) was at least 1.25 ng/test.

### Precision evaluation of triplex amplification analysis

**Figure 3:**
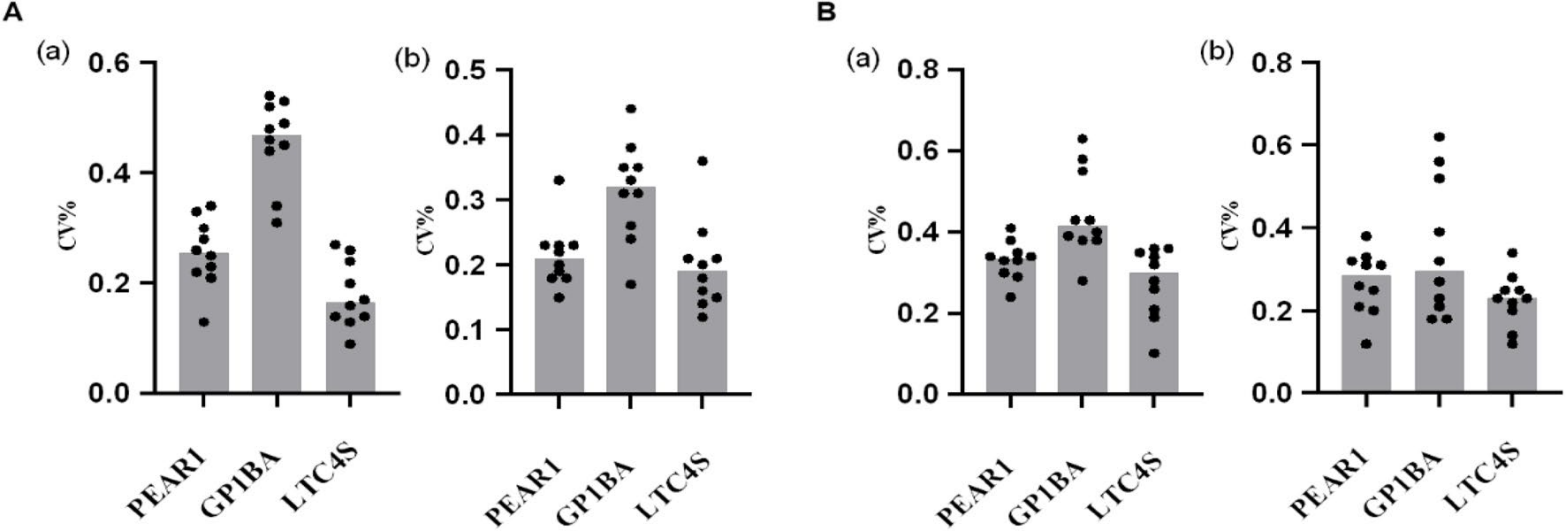
Intra-day coefficient of variant (CV) for PEAR1 (rs12041331), GP1BA (rs6065) and LTC4S (rs730012). Genomic DNA at 10 ng (**A**) or 2.5 ng (**B**) level was measured in eight plicates by two operators with two reagent lots every day over 5 days (n = 80/specimen) at one site. Data were expressed as CV median in wild (**a**) or mutated-type (**b**) forward primer-based amplification assay.

The precision of the triplex amplification analysis was evaluated by detection of genomic DNA at 10 ng/test and 2.5 ng/test levels. Each specimen was tested in eight plicates by two operators with two reagent lots every day over 5 days (n = 80/specimen) at one site. A total of eighty Cq values were collected to calculate the coefficient of variance (CV). The results revealed that CV value was < 2% for all days, specimens, replicates, operators and reagent lots combined. Figure 3 shows the intra-day CV for PEAR1(rs12041331), GP1BA (rs6065) and LTC4S (rs730012).

### Agreement analysis between triplex amplification analysis and Sanger sequencing

We conducted the triplex amplification analysis on each of 189 samples, in which 89, 165, and 136 specimens were defined as homozygotes for rs12041331 (PEAR1), rs6065 (GP1BA) and rs730012 (LTC4S), respectively, while 100, 24, and 53 samples were defined as heterozygotes for rs12041331 (PEAR1), rs6065 (GP1BA) and rs730012 (LTC4S), respectively. The cut-off values for genotyping were described in Table 3.

**Table 3:** The cut-off value for genotyping.

Genetic polymorphism	wild-type homozygote	mutated-type homozygote	Heterozygote
PEAR1, rs12041331	∆Cq = (mCq − wCq) > 2	∆Cq = (wCq − mCq) > 2	∆Cq = ∣ wCq − mCq ∣ ≤ 2
GP1BA, rs6065	∆Cq = (mCq − wCq) > 1	∆Cq = (wCq − mCq) > 1	∆Cq = ∣ wCq − mCq ∣ ≤ 1
LTC4S, rs730012	∆Cq = (mCq − wCq) > 1	∆Cq = (wCq − mCq) > 1	∆Cq = ∣ wCq − mCq ∣ ≤ 1

wCq: Cq value in wild-type forward primer-based amplification assay; mCq: Cq value in mutated-type forward primer-based amplification assay

Pharmacogenomics appears that some SNP_s_ are more likely to initiate AR and adverse reactions^36,37^. Behaving as a kind of platelet transmembrane protein, the platelet endothelial aggregation receptor 1 (PEAR1) plays an important role in platelet aggregation. And genetic polymorphism of the rs12041331 in PEAR1 gene can obviously affect the inhibitive effect of aspirin on platelet aggregation^38^. Glycoprotein Ib-alpha (GP1BA) gene encodes platelet surface membrane glycoprotein (GPIb) that is a heterodimer consisting of bisulfide-linked α and β subunits, and acts as a receptor for von Willebrand factor(VWF)^39^. Genetic polymorphism of the rs6065 in GP1BA gene was evidenced to correlate aspirin resistance^40,41^. It was documented that patient carrying C-type allele for rs730012 in leukotriene C4 synthase (LTC4S) gene are prone to aspirin-induced urticaria^42,43^. Consulting to a scoring table proposed by Guangdong Pharmaceutical Association (Guangzhou, China)^32^ (Table 4), a triplex amplification analysis to detect genetic polymorphism of gDNA rs12041331 (PEAR1), rs6065 (GP1BA) and rs730012 (LTC4S) was designed and substantiated in this study. The results of the agreement analysis indicated the genotyping outlined by the triplex amplification analysis is consistent with the results obtained from Sanger sequencing.

**Table 4:** Pharmacogenomics guidelines for personalized aspirin delivery^32^.

Genetic polymorphism	Phenotype scoring	Scoring grade and individualized advice
PEAR1 rs12041331, G > A	GG: 2 points GA: 1 point AA: 0 point	Aspirin resistance 0 point: low response, recommended drug change or single-use dose 200 mg or more based on platelet function test 1 point: intermediate response, recommended single-use dose 150 mg 2–4 points: high response, recommended single-use dose 75–100 mg
GP1BA rs6065, C > T	CC: 0 points CT: 1 point TT: 2 points	
LTC4S rs730012, A > C	AA: 0 points AC: 1 point CC: 2 points	Adverse reaction 0 point: low risk, medication safe, no prompts 1 point: certain risk, this suggests some risk of an allergic reaction and the patient is instructed to be closely observed 2 points: high risk, it is recommended to pay close attention to the risk of adverse reactions or switch to other drugs if they occur

In summary, we established a simple, efficient and accurate approach to the determination of genetic polymorphism of gDNA rs12041331 (PEAR1), rs6065 (GP1BA) and rs730012 (LTC4S), which can be used to guide aspirin delivery to reduce AR and adverse reaction.

## Data Availability

Primer and probe sequences related to this study are available within this manuscript. The primers and probes were designed in accordance with the gene sequences of rs12041331(PEAR1), rs6065 (GP1BA) and rs730012 (LTC4S), which are available in NCBI database. Raw data are available from the corresponding authors upon reasonable request.
